# An analysis of national target groups for monovalent 2009 pandemic influenza vaccine and trivalent seasonal influenza vaccines in 2009-10 and 2010-11

**DOI:** 10.1186/1471-2334-11-230

**Published:** 2011-08-26

**Authors:** Sophia Ng, Peng Wu, Hiroshi Nishiura, Dennis KM Ip, Esther ST Lee, Benjamin J Cowling

**Affiliations:** 1 School of Public Health, Li Ka Shing Faculty of Medicine, The University of Hong Kong, Pokfulam, Hong Kong Special Administrative Region, China; 2 PRESTO, Japan Science and Technology Agency, Saitama 332-0012, Japan

**Keywords:** influenza, pandemic, seasonal, vaccine, target groups, subsidy

## Abstract

**Background:**

Vaccination is generally considered to be the best primary prevention measure against influenza virus infection. Many countries encourage specific target groups of people to undertake vaccination, often with financial subsidies or a priority list. To understand differential patterns of national target groups for influenza vaccination before, during and after the 2009 influenza pandemic, we reviewed and analyzed the country-specific policies in the corresponding time periods.

**Methods:**

Information on prioritized groups targeted to receive seasonal and pandemic influenza vaccines was derived from a multi-step internet search of official health department websites, press releases, media sources and academic journal articles. We assessed the frequency and consistency of targeting 20 different groups within populations which are associated with age, underlying medical conditions, role or occupations among different countries and vaccines. Information on subsidies provided to specific target groups was also extracted.

**Results:**

We analyzed target groups for 33 (seasonal 2009 and 2009-10 vaccines), 72 (monovalent pandemic 2009-10 vaccine) and 34 (seasonal 2010 and 2010-11 vaccines) countries. In 2009-10, the elderly, those with chronic illness and health care workers were common targets for the seasonal vaccine. Comparatively, the elderly, care home residents and workers, animal contacts and close contacts were less frequently targeted to receive the pandemic vaccine. Pregnant women, obese persons, essential community workers and health care workers, however, were more commonly targeted. After the pandemic, pregnant women, obese persons, health care and care home workers, and close contacts were more commonly targeted to receive the seasonal vaccine compared to 2009-10, showing continued influence from the pandemic. Many of the countries provided free vaccines, partial subsidies, reimbursements or national health insurance coverage to specific target groups and over one-third of the countries offered universal subsidy regarding the pandemic vaccine. There was also some inconsistency between countries in target groups.

**Conclusions:**

Differences in target groups between countries may reflect variable objectives as well as uncertainties regarding the transmission dynamics, severity and age-specific immunity against influenza viruses before and after vaccination. Clarification on these points is essential to elucidate optimal and object-oriented vaccination strategies.

## Background

Influenza vaccinations are effective in preventing confirmed influenza virus infections in infants [1], children [2] and healthy adults [3]. However, the elderly are thought to face the highest risk of severe influenza-associated illness in interpandemic years [4,5], and influenza vaccinations are often recommended to this group although their effectiveness is uncertain [6]. In addition to targeting specific age groups, health authorities also commonly identify other target groups based on characteristics such as underlying medical conditions or occupations that may place people at higher risk of infection, higher risk of severe disease if infected, or higher risk of transmission to other vulnerable people. To facilitate uptake in these target groups, priority access and subsidies to receive influenza vaccines are often provided to them.

Every year, the World Health Organization (WHO) issues recommendations on influenza vaccine composition in February for the Northern Hemisphere, and September for the Southern Hemisphere. It usually takes around 6 months after the recommendation meeting for the vaccines to become available. In April 2009, a novel influenza A(H1N1) virus emerged in North America and rapidly spread around the world in the first influenza pandemic of the 21^st ^century. Early in the pandemic it was reported that existing seasonal influenza vaccinations including the 2009-10 trivalent seasonal influenza vaccine were not likely to provide substantial protection against the pandemic H1N1 (pH1N1) virus, while the prevalence of pre-existing cross-reactive antibody against pH1N1, likely indicative of some degree of protection against infection, was very low in most age groups except the elderly [7]. Monovalent pH1N1 vaccines became available around the same time as the 2009-10 trivalent seasonal influenza vaccine towards the end of 2009. The pH1N1 virus subsequently replaced the seasonal A(H1N1) virus in the 2010 trivalent seasonal influenza vaccine in the Southern Hemisphere, and the 2010-11 trivalent seasonal influenza vaccine in the Northern Hemisphere.

The pH1N1 virus appears to have displaced the pre-pandemic seasonal influenza A(H1N1) viruses in global circulation, while in some countries seasonal A(H3N2) and B viruses also have continued to circulate. Influenza vaccination remains the best primary preventive measure against these viruses. In addition to pre-pandemic studies that took place prior to 2009 [8,9], the target groups for influenza vaccinations before and during the pandemic have been studied in Europe and the Americas [10-12]. To investigate differential patterns of vaccination targets and subsidy by different vaccines and countries, we report and compare groups targeted in different regions of the world to receive the monovalent pH1N1 vaccine, the 2009 (Southern Hemisphere) and 2009-10 (Northern Hemisphere) seasonal vaccines which included a seasonal A/Brisbane/59/2007(H1N1)-like virus, and the post-pandemic seasonal vaccines in 2010 and 2010-11 that included a A/California/7/2009 (H1N1)-like (pH1N1) virus.

## Methods

We grouped together the 2009 (Southern Hemisphere) and 2009-10 (Northern Hemisphere) seasonal vaccines as "S0910" vaccines since they both had the same virus composition determined before the pandemic. Similarly "S1011" vaccines referred to the 2010 (Southern Hemisphere) and 2010-11 (Northern Hemisphere) seasonal vaccines which had the same composition including the pH1N1 virus. We used P0910 vaccine as the abbreviation for the 2009 monovalent pH1N1 vaccine. All these vaccines were either adjuvanted or unadjuvanted, including whole or split virions. Target groups were identified as those that were explicitly listed as prioritized in each country to receive each of the vaccines through a structured search of online resources. Since P0910 competed for time compared to S0910 and S1011, and because the total number of prioritized groups varied substantially, the order of prioritization was not considered in the present study. In addition we searched for information on subsidies provided to specific target groups.

### Search strategy

Information on the groups targeted to receive the seasonal and pandemic influenza vaccines was obtained by a multistep internet search performed from August 2010 through March 2011. Based on a list of 211 countries [13], the official website of the national health authorities were located using search terms < country name > AND ("health ministry" OR "health department") using http://google.com, http://bing.com and http://yahoo.com. A combination of search terms ("influenza" OR "pandemic" OR "seasonal") AND ("2009" OR "2010" OR "2011") AND ("vaccin*" OR "immunization" OR "immunisation") were then entered to the search engine embedded in the official website. When no relevant results were found, electronic press releases of the national health authorities dated from July 2009 through January 2011 were manually screened. Direct searches using < country name > and the above combination of search terms were performed if relevant information was not retrievable from the official websites. We regarded electronic news and academic journal articles as acceptable secondary sources. The search terms were translated into the official language of the country using Google translate http://translate.google.com when appropriate results were not found using English search terms. The information and the sources relevant to the priority and subsidy groups were extracted into standardized forms.

### Analysis

After reviewing the reported target groups, we classified them into three broad types, namely those defined by age, those related to underlying medical conditions, and those related to role or occupation. Regarding age-based target groups, exact age brackets varied from country to country. To facilitate comparison we categorized target ages into 6 age groups: *0-5 years *(pre-school children), *6-11 years *(junior school children), *12-15 years *(high school children), *16-39 years *(young adults), *40-64 years *(middle-aged adults) and *65 years or over *(elderly).

We defined 4 groups relating to underlying medical conditions. We defined *pregnant (prg) *to include woman at any trimester of pregnancy, although some countries targeted women only in the third (or second and third) trimesters. *Chronic illness (chr) *included individuals with any chronic non-communicable and communicable conditions. This group also included individuals with HIV/AIDS, immunosuppression and pediatric conditions requiring long-term acetylsalicylic acid therapy. *Obesity (obe) *was typically defined as having body mass index of ≥ 40 kg/m^2^. *Disabled (dis) *individuals included those with mental or physical disabilities.

We defined 10 groups relating to specific roles or occupations. *Health care workers (hcw) *were health professionals and personnel involved with patient care in hospital and ambulatory settings. *Laboratory workers (lab) *were those with potential contact with specimens containing or contaminated with influenza viruses. *Close contacts of high-risk groups (con) *included those persons, whether paid or unpaid, who have close contact with or who take care of individuals at risk of severe illness if infected, some of whom may not be able to receive influenza vaccine themselves, in households, day care centers or other institutions except for long-term care facilities, hospital and ambulatory settings. *Care home residents (res) *and *workers (chw) *included residents and workers in long-term care facilities for the elderly and other persons at risk of influenza infection. *Teachers (tch) *were persons who provide formal education to children in a school setting. *Animal contacts (ani) *referred to those with direct contact with animals that could be potential hosts of influenza viruses, in farms, markets, abattoirs or during culling operations. *Essential community workers (ecw) *referred to persons providing services that are essential to the operation of the country, including key administrative personnel, police, firemen, customs officer, military personnel, etc. *Aboriginals (abo) *referred to indigenous persons and *travelers (trv) *included frequent travelers, persons travelling to areas with sustained transmission, and pilgrims.

After classifying target groups into the above categories, we visually presented target groups for each vaccine and compared patterns for different vaccines and between countries. Subsidy groups were categorized similarly as the target groups with addition of the *financial need *group for whose low-income cannot afford the vaccine and *universal *for countries which provided subsidy for all. The proportion of observed agreement (by different vaccines or between countries) was investigated to characterize targeting patterns of prioritization and subsidy. Moreover, as an objective measurement of consistency in each prioritized group between vaccines and between countries, we employed a multiple-rater agreement coefficient, the AC_1 _statistic [14]. AC_1 _is known as a new measurement of consistency, replacing unstable kappa coefficient due to the paradox-resistant robustness [14]. The AC_1 _statistic can take any value from -1 to 1, with 1 indicating full consistency, 0 absence of consistency (the degree of agreement expected by chance), and negative values indicating inconsistency or disagreement. To analyze differences in target groups, AC_1 _was computed for each pair of two different vaccines, and between countries for each vaccine. The 95% confidence intervals for the AC_1 _statistics were obtained using bootstrap resampling with 1,000 resamples. We used χ^2 ^and Fisher's exact tests to compare target groups for the P0910 vaccine between countries that did or did not receive donated vaccines via the WHO.

## Results

**Figure 1:**
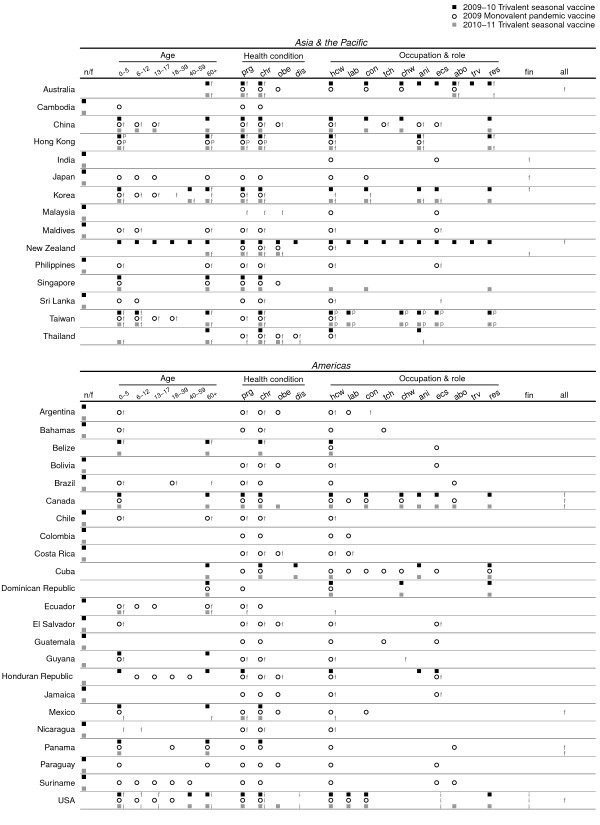
**Groups prioritized to receive the 2009 and 2009-10 seasonal trivalent influenza vaccine (black square), 2009 monovalent pandemic influenza vaccine (black open circle), and 2010 and 2010-11 seasonal trivalent influenza vaccine (gray square) in countries located in Asia and the Pacific, and the Americas**. Groups subsidized to receive the vaccine for free (f), at a cost with partial subsidy (p), with reimbursable cost (r) or insured by national health insurance (i) were also denoted. Target group abbreviations: *0-5 *(0-5 years), *6-11 *(6-11 years), *12-15 *(12-15 years), *16-39 *(16-39 years), *40-64 *(40-64 years), *65+ *(65 years or over), *prg *(pregnant), *chr *(chronic illness, *obe *(obesity), *dis *(disabled), *hcw *(health care workers), *lab *(laboratory workers), *con *(close contacts), *res *(care home residents), *chw *(care home workers), *tch *(teachers), *ani *(animal contacts), *ecw *(essential community workers), *abo *(aboriginals), *trv *(travelers).

**Figure 2:**
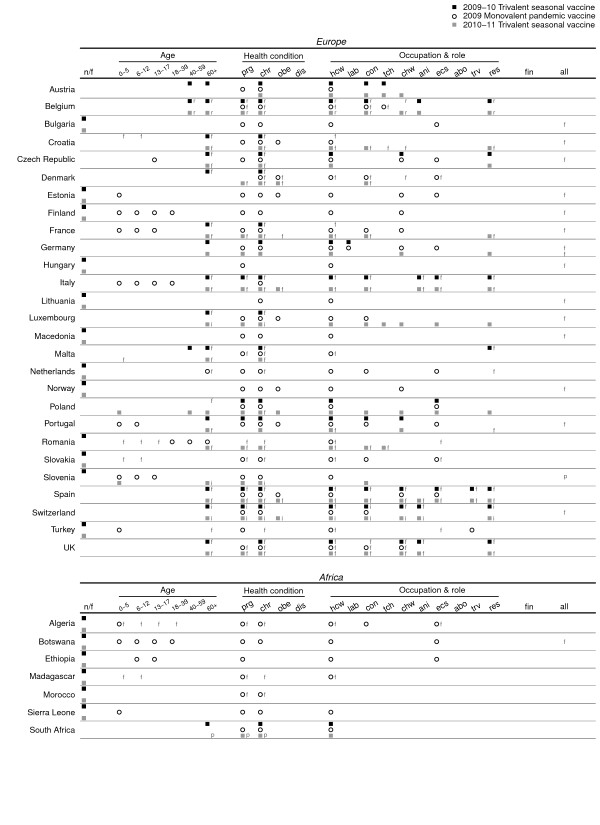
**Groups prioritized to receive the 2009 and 2009-10 seasonal trivalent influenza vaccine (black square), 2009 monovalent pandemic influenza vaccine (black open circle), and 2010 and 2010-11 seasonal trivalent influenza vaccine (gray square) in countries located in Europe and Africa**. Groups subsidized to receive the vaccine for free (f), at a cost with partial subsidy (p), with reimbursable cost (r) or insured by national health insurance (i) were also denoted. Abbreviations for age, underlying medical conditions, role and occupation are the same as those employed in Figure 1.

**Figure 3:**
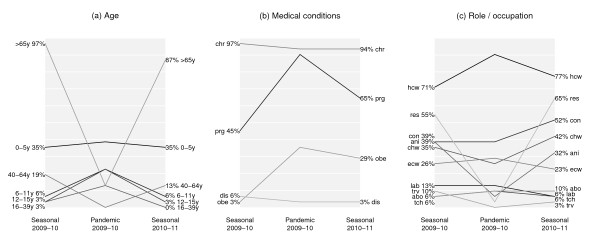
**The frequency of risk groups prioritized to receive the seasonal trivalent influenza vaccine, 2009 monovalent pandemic influenza vaccine, and 2010 and 2010-11 trivalent seasonal influenza vaccine in 31 countries in which the target group information were found for all 3 vaccines**. Abbreviations for age, underlying medical conditions, role and occupation are the same as those employed in Figure 1. White grid lines are included at 10% intervals.

**Figure 4:**
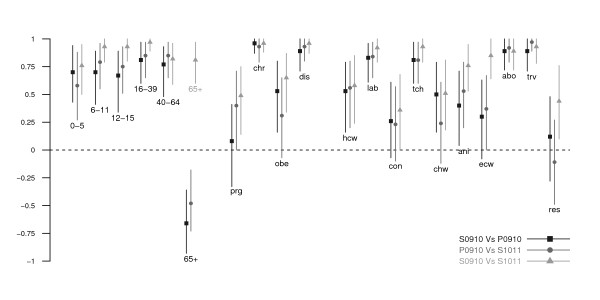
**The pairwise consistency of target groups between the 2009 and 2009-10 trivalent seasonal influenza vaccine, the 2009-10 monovalent pandemic influenza vaccine and the 2010 and 2010-11 trivalent seasonal influenza vaccines**. Points show the estimates of the multiple-rater agreement AC_1 _statistics, lines show the bootstrap-based 95% confidence intervals. Abbreviations for age, underlying medical conditions, role and occupation are the same as those employed in Figure 1.

**Figure 5:**
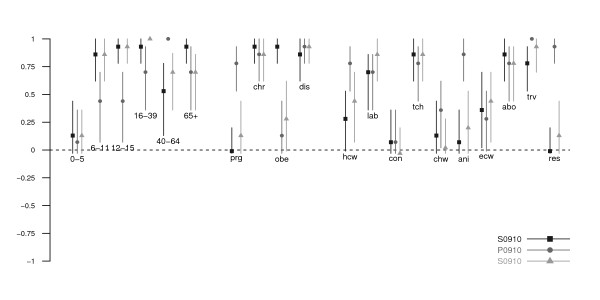
**The consistency of target groups between the 31 countries in which the target group information were found for all 3 vaccines**. Points show the estimates of the multiple-rater agreement AC_1 _statistics, lines show the bootstrap-based 95% confidence intervals. Abbreviations for age, underlying medical conditions, role and occupation are the same as those employed in Figure 1.

Recommendations on the priority and subsidy groups were found for 72 and 57 countries relevant to the P0910 vaccine; 33 and 20 countries for the S0910 vaccine; 34 and 28 countries for the S1011 vaccine. The majority of the information was retrieved from national health authority websites, and full details are provided in Additional File 1. The countries with retrieved information on vaccination were distributed in Africa, the Americas, Asia and the Pacific, and Europe. Sixteen of these countries received donations of monovalent pH1N1 vaccine via the WHO [15]. Subsidy for vaccination was provided in various ways in different countries. Some of the countries provided the vaccine for free without any charge to the recipient, or at a cost in which part of the vaccine and administration fee were subsidized. In several places, the vaccines were covered in the national health insurance programs for specific risk groups (Figures 1 and 2, Additional File 1). The definition of chronic illness varied between countries. The detailed lists of health conditions included by each country are shown in Additional File 1. Priority and subsidy groups in individual countries in Asia, the Pacific and the Americas are shown in Figure 1, and those for Europe and Africa are shown in Figure 2. To allow for comparison of target groups between vaccines, Figure 3 shows the proportions of observed agreement for each target group among 31 countries where information on all 3 vaccines was identified (the 31 countries included in Figure 3 corresponded to those without "n/f" indicated in Figure 1 and 2). Figures 4 and 5 compare the consistency of target groups between vaccines and between countries using AC_1 _statistics, respectively.

### Target groups for the 2009 and 2009-10 seasonal vaccine

Almost all the countries prioritized the elderly (97%) and those with chronic illness (91%) to receive the S0910 vaccine (Figures 1 and 2, Table 1). Health care workers (70%) and care home residents (52%) were targeted by over half of the countries. Pregnant women were not consistently targeted (46%) and very few countries targeted the obese (3%). Like the priority groups, many countries provided subsidy to the elderly (90%), those with chronic illnesses (80%), health care workers (45%) and care home residents (45%) to receive the vaccine. Pregnant women were not always subsidized (30%). Countries included in our study were not as consistent in including in target groups children aged 0-5 years, pregnant women, close contacts, animal contacts, care home residents and workers (Figure 5).

### Target groups for the 2009 monovalent pH1N1 vaccine

Pregnant women, health care workers and those with chronic illnesses were commonly targeted (~90%). Around 50% of the countries targeted pre-school children, one-fifth to one-fourth for older children, obese persons and close contacts. Compared to the S0910 vaccine, elderly, care home residents and workers, close contacts, and animal contacts were less commonly targeted to receive P0910 vaccine (Figures 1, 2 and 3, Table 1). More commonly targeted groups included children of all age, young adults, pregnant women, obese persons, essential community workers and health care workers. The low AC_1 _values among these groups indicate that the target groups between two vaccines were inconsistent (Figure 4). The AC_1 _value was lowest in the comparison for the elderly group between P0910 and either seasonal vaccine. The AC_1 _values of these two combinations (i.e. comparisons between pandemic and either of seasonal vaccines) for pregnant women and care home residents were also very low and close to 0. Among countries that received donation of P0910 vaccine via the WHO, all of them had targeted pregnant women (100%), and most of them had recommended those with chronic illnesses (94%) and health care workers (94%) to receive vaccination (Additional File 2). Within these countries, pre-school children were the most commonly targeted age group.

**Table 1:** Frequency of target groups prioritized and subsidized to receive the pandemic 2009-10, seasonal 2009 & 2009-10, and seasonal 2010 & 2010-11 vaccines

	Seasonal 2009-10 vaccine				Pandemic 2009-10 vaccine				Seasonal 2010-11 vaccine			
	
	Priority		Subsidy		Priority		Subsidy		Priority		Subsidy	
	n	(%)	n	(%)	n	(%)	n	(%)	n	(%)	n	(%)
**Countries**	33	-	20	-	72	-	57	-	34	-	28	-
												
**Age (year)**												
0-5	13	(39.4)	5	(25.0)	33	(45.8)	18	(31.6)	13	(38.2)	8	(28.6)
6-11	2	(6.1)	3	(15.0)	17	(23.6)	9	(15.8)	2	(5.9)	2	(7.1)
12-15	1	(3.0)	1	(5.0)	15	(20.8)	5	(8.8)	1	(2.9)	1	(3.6)
16-39	1	(3.0)	0	(0.0)	10	(13.9)	4	(7.0)	0	(0.0)	0	(0.0)
40-64	6	(18.2)	1	(5.0)	3	(4.2)	0	(0.0)	4	(11.8)	2	(7.1)
65+	32	(97.0)	18	(90.0)	12	(16.7)	9	(15.5)	30	(88.2)	23	(82.1)
												
**Underlying medical conditions**												
Chronic illness	30	(90.9)	16	(80.0)	63	(87.5)	31	(54.4)	31	(91.2)	22	(78.6)
Pregnant	15	(45.5)	6	(30.0)	65	(90.3)	26	(45.6)	22	(64.7)	16	(57.1)
Disabled	2	(6.1)	1	(5.0)	1	(1.4)	1	(1.8)	1	(2.9)	2	(7.1)
Obese	1	(3.0)	0	(0.0)	20	(27.8)	7	(12.3)	9	(26.5)	7	(25.0)
												
**Role & occupation**												
Health care worker	23	(69.7)	9	(45.0)	66	(91.7)	27	(47.4)	25	(73.5)	14	(50.0)
Care home resident	17	(51.5)	9	(45.0)	1	(1.4)	1	(1.8)	20	(58.8)	11	(39.3)
Animal contact	13	(39.4)	5	(25.0)	2	(2.8)	2	(3.5)	10	(29.4)	8	(28.6)
Close contact	12	(36.4)	5	(25.0)	16	(22.2)	5	(8.8)	18	(52.9)	10	(35.7)
Care home worker	11	(33.3)	5	(25.0)	11	(15.3)	5	(5.3)	13	(38.2)	6	(21.4)
Essential community service	9	(27.3)	4	(20.0)	27	(37.5)	13	(22.8)	7	(20.6)	5	(17.9)
Laboratory worker	4	(12.1)	1	(5.0)	7	(9.7)	1	(1.8)	2	(5.9)	1	(3.6)
Traveler	3	(9.1)	1	(5.0)	1	(1.4)	0	(0.0)	1	(2.9)	1	(3.6)
Teacher	2	(6.1)	0	(0.0)	5	(6.9)	2	(3.5)	3	(8.8)	2	(7.1)
Aboriginal	2	(6.1)	1	(5.0)	5	(6.9)	0	(0.0)	3	(8.8)	1	(3.6)
												
Financial need*	-	-	2	(10.0)	-	-	3	(5.3)	-	-	2	(7.1)
												
**Universal^†^**	-	-	2	(10.0)	-	-	21	(36.8)	-	-	3	(10.7)
												

* *financial need *group for whose low-income cannot afford the vaccine.

**^† ^***universal *for countries which provided subsidy for all.

Unlike the S0910 vaccine, the elderly (16%), care home residents (2%) and workers (5%) were not often subsidized to receive the P0910 vaccine. Contrarily, children especially those aged below 5 years (32%) and pregnant women (46%) were commonly subsidized. Similar to the S0910 vaccine, many countries subsidized those with chronic illnesses (54%) and health care workers (47%) to receive the P0910 vaccine. Young adults (7%) and the obese (12%) had not been subsidized prior to the P0910 vaccine. While most of the countries only offered subsidy to the targeted groups for the seasonal vaccines, 21/57 countries provided universal subsidy to their population for the pandemic one. The countries showed higher consistency in determining whether to target the middle age, pregnant women, health care workers, care home residents and workers, animal contacts and travelers to receive the P0910 compared to the seasonal vaccines (Figure 5). There was less consistency in targeting the younger age groups, the elderly and obese persons.

### Target groups for the 2010 and 2010-11 trivalent seasonal vaccine

The relative frequencies of groups prioritized for the S1011 vaccine were generally similar to the S0910 vaccine (Figures 1, 2 and 3, Table 1) with several notable changes. One particular change following the pandemic was that pregnant women and obese persons were more commonly targeted for the S1011 vaccine than they had been for the S0910 vaccine. Interestingly, close contacts, health care workers and care home workers were also more frequently targeted for the seasonal vaccine after the pandemic. As mentioned above, some groups including the elderly, care home residents, and animal contacts were less commonly targeted for the P0910 vaccine compared to the S0910 vaccine. In 2010-11, these groups continued to be targeted as in the previous year for the S0910 vaccine. This was also reflected by the higher AC_1 _values when S1011 is compared against S0910 vaccine (Figure 4). The pandemic also appeared to have reduced the consistency to target the elderly and obese persons for S1011 vaccine among different countries (Figure 5).

Like the S0910 vaccine, the elderly (82%), those with chronic illnesses (79%), health care workers (50%) and care home residents (39%) were frequently subsidized to receive the S1011 vaccine. Pregnant women (57%) and the obese (25%) continued to be subsidized after becoming new target groups for the P0910 vaccine. Close contacts (36%) not only were more frequently prioritized, but they were also subsidized by many countries to receive the S1011 vaccine.

## Discussion

There were some notable differences between groups targeted for the 2009-10 pandemic and seasonal influenza vaccines, and substantial differences in target groups between countries. The greatest inconsistency in target groups was seen in comparisons between S0910 and P0910. Some groups targeted for the pandemic vaccine more commonly than for the 2009-10 seasonal vaccine, including pregnant women and obese persons, and health care workers were subsequently more commonly targeted for the 2010-11 post-pandemic seasonal vaccine. Subsidies to increase vaccine uptake were available to target groups in many countries by either providing the vaccine for free, at a subsidized cost or through national health insurance (Figures 1 and 2).

Before further discussing the patterns in target groups, two important issues should be noted: (A) the social and political backgrounds of policy decisions to prioritize certain groups for P0910 are likely to differ from those for seasonal vaccines and (B) the 'best' or 'optimal' strategy to achieve certain public health objectives has yet to be fully clarified in the context of including or excluding certain groups for prioritization. As for (A), compared to seasonal vaccines, public health decision of prioritized groups for P0910 may be more associated with maintaining social security during emergency vaccination and avoiding any confusion that could arise from 'first come, first served' basis among the public [8]. On the other hand, policymaking of seasonal vaccines is unlikely to face needs to give rapid decisions for implementing emergency vaccination programmes and to maintain social security, and rather, target groups of seasonal vaccines may sometimes reflect the results of surveys of public demands among the potential target groups and may also take into account aspects of cost-benefit and cost-effectiveness. With respect to (B), various studies including modeling exercises considered optimal vaccination strategies [16-18] but those exercises have not allowed full clarification of all target groups. It should be emphasized that the limitations of modeling studies and inconsistency in target groups (as seen in the present study) may be attributable to non-uniform public health objectives of vaccination, such as reducing peak hospital burden versus reducing overall mortality, as well as uncertainties with respect to the transmission dynamics, severity and age-specific immunity against influenza viruses before and after vaccination. The objective of this study has been not to criticize certain choices of target groups, but to explore to what extent target groups are similar between countries, or even within countries from year to year and between the seasonal and pandemic vaccines.

Some inconsistent patterns were seen between pandemic and seasonal vaccines in targeting certain age groups, those with underlying medical conditions and those with occupations associated with higher risk. First, mathematical modeling studies have shown that achieving high vaccination coverage among school-age children could substantially elevate herd immunity and protect other risk groups (including elderly and those with underlying diseases) by means of a 'transmission-limiting' strategy [17-20]. Nevertheless, many countries experienced substantial attack rates in children before vaccines became available [21], and in such an instance (e.g. after observing epidemic peak), effectiveness of transmission-limiting strategy tends to be minimal [19,22]. While school-age children were targeted for the P0910 vaccine in some countries, most countries included in our review adopted a morbidity-limiting strategy and targeted groups at high risk of severe disease (Figures 1 and 2). Vaccine effectiveness in the elderly remains uncertain [6] but the elderly are commonly targeted to receive interpandemic influenza vaccine because of their high risk of severe disease [4,5]. However, early seroepidemiological studies in 2009 suggested that the elderly may be protected against infection [23], and few countries included elderly in the target groups for the P0910 vaccine (Figure 3 and Table 1).

Second, with regard to underlying medical conditions, pregnant and postpartum women appeared to be at higher risk of severe disease if infected from the early stages of the pandemic [24-27] and many countries prioritized this group to receive the vaccine when it became available. Whereas pregnant women were less commonly targeted for seasonal influenza vaccination before the pandemic, evidence of an increased risk of severe influenza had already been observed in pregnant women before 2009 [28-30]. Influenced by the 2009 pandemic, 22/34 countries targeted this group for S1011 vaccine (Figures 1 and 2, Table 1). Other than pregnant women, a number of studies during the pandemic found obesity to be a risk factor for hospitalization and death [26,31-33], and some countries incorporated obese individuals into the target groups for the P0910 vaccine and again retained them in the target groups for the post-pandemic S1011 vaccine (Figures 1 and 2, Table 1).

Third, regarding roles and occupations, health care workers were targeted for both pandemic and seasonal influenza vaccines by many countries and they have been increasingly targeted during and after the course of the pandemic (Figures 1, 2 and 3, Table 1), although they may not necessarily face higher risk of infection due to occupational exposures [34,35] and the issue of mandatory vaccination in this group remains controversial [36,37]. Workers in long term residential care homes were less commonly targeted for the pandemic vaccine compared to the seasonal vaccines, perhaps because of the apparent low risk of pH1N1 in the elderly [7,21]. Animal contacts might have been less commonly targeted merely because of displacement by other risk groups as P0910 vaccine was initially expected to be scarce. Later they were more commonly targeted for the S1011 influenza vaccine which included the pandemic strain.

A few limitations should be discussed. We were not able to find information on priority groups in many countries, and thus, the data included in our review may not sufficiently represent the worldwide patterns in target groups. In addition, information available online could differ from what was actually implemented, even though most of the information included in our study was from official government websites. Potentially lower quality sources were excluded by limiting our search to official webpage of the government, news or academic journal articles. Future studies of influenza vaccine policies could consider a survey-based approach interviewing a representative health official from each country. Actual vaccine uptake or influenza-associated morbidity should also be investigated in relation to the policy decisions of target groups when relevant data are available as part of policy evaluations. There were also within country variations; e.g. recommendations on target groups in Canada and the United States differed between states. In these situations, the federal recommendations were used to reflect the central policy, but more detailed investigation of the heterogeneity in target groups between states or regions could be a topic for further research especially for a within-country policy evaluation.

## Conclusions

Countries selected different target groups for the pandemic vaccine and seasonal vaccines before and after the pandemic. Our results also highlighted substantial inconsistencies between individual countries for each vaccine. Although inconsistency between vaccines partly represents the influence of differential social backgrounds of policymaking, the inconsistencies essentially highlight differential public health objectives of vaccination and the ongoing uncertainty with respect to the transmission dynamics, severity profile and immunology of influenza. Improving our understanding of influenza epidemiology must help optimize vaccination strategies in the future.

## Abbreviations

WHO: World Health Organization

## Competing interests

BJC reports receiving research grant funding from MedImmune Inc., a manufacturer of influenza vaccines. DKMI received a research grant from Hoffmann-La Roche Ltd., a manufacturer for antiviral drugs for influenza. The authors report no other potential conflicts of interest.

## Authors' contributions

The study was designed by SN, HN, EL and BJC. The searches for online resources were conducted by SN and PW. All authors contributed to the analysis and interpretation of data, have been involved in drafting the manuscript or revising it critically for important intellectual content, and have given final approval of the version to be published.

## Pre-publication history

The pre-publication history for this paper can be accessed here:

http://www.biomedcentral.com/1471-2334/11/230/prepub

## Supplementary Material

Additional file 1 **Detailed summary of priority information**. Detailed data on policies in different countries for the monovalent 2009 pandemic influenza vaccine and the pre- and post-pandemic trivalent seasonal influenza vaccines. Click here for file

Additional file 2 **Priority groups in countries that received donated vaccine**. Comparison of priority groups in countries that received or did not receive donation of 2009-10 monovalent pandemic vaccine via the World Health Organization. Click here for file
