# Supplementary material for: An analysis of national target groups for monovalent 2009 pandemic influenza vaccine and trivalent seasonal influenza vaccines in 2009-10 and 2010-11
Source: BMC Infect Dis. 2011 Aug 26;11:230. doi: 10.1186/1471-2334-11-230 (PMC3175216; doi:10.1186/1471-2334-11-230)
Supplement: Additional file 2 — Priority groups in countries that received donated vaccine. Comparison of priority groups in countries that received or did not receive donation of 2009-10 monovalent pandemic vaccine via the World Health Organization. [file 1471-2334-11-230-S2.DOC]

Table S2. Priority groups in countries that received or did not receive donation of 2009-10 monovalent pandemic vaccine via the World Health Organization.

|  | Received P0910 vaccine donation via WHO (16 countries*) | |  | Did not receive P0910 vaccine donation via WHO (56 countries) | |  | p-value^†^ |
| --- | --- | --- | --- | --- | --- | --- | --- |
|  | n | (%) |  | n | (%) |  |  |
| **Age group** |  |  |  |  |  |  |  |
| 0-5 y | 11 | (68.8) |  | 22 | (39.3) |  | 0.05 |
| 6-11 y | 5 | (31.2) |  | 12 | (21.4) |  | 0.51 |
| 12-15 y | 3 | (18.8) |  | 12 | (21.4) |  | 1.00 |
| 16-39 y | 3 | (18.8) |  | 7 | (12.5) |  | 0.68 |
| 40-64 y | 2 | (12.5) |  | 1 | (1.8) |  | 0.12 |
| 65+ y | 4 | (25.0) |  | 8 | (14.3) |  | 0.45 |
|  |  |  |  |  |  |  |  |
| **Underlying medical conditions** |  |  |  |  |  |  |  |
| Pregnant | 16 | (100.0) |  | 49 | (87.5) |  | 0.34 |
| Chronic illness | 15 | (93.8) |  | 48 | (85.7) |  | 0.67 |
| Obese | 4 | (25.0) |  | 16 | (28.6) |  | 1.00 |
| Disabled | 0 | (0.0) |  | 1 | (1.8) |  | 1.00 |
|  |  |  |  |  |  |  |  |
| **Role & occupation** |  |  |  |  |  |  |  |
| Health care worker | 15 | (93.8) |  | 51 | (91.1) |  | 1.00 |
| Essential community  service | 9 | (56.2) |  | 18 | (32.1) |  | 0.09 |
| Laboratory worker | 1 | (6.2) |  | 6 | (10.7) |  | 1.00 |
| Close contact | 1 | (6.2) |  | 15 | (26.8) |  | 0.10 |
| Teacher | 1 | (6.2) |  | 4 | (7.1) |  | 1.00 |
| Care home worker | 1 | (6.2) |  | 10 | (17.9) |  | 0.44 |
| Care home resident | 1 | (6.2) |  | 0 | (0.0) |  | 0.22 |
| Aboriginal | 1 | (6.2) |  | 4 | (7.1) |  | 1.00 |
| Animal contact | 0 | (0.0) |  | 2 | (3.6) |  | 1.00 |
| Traveler | 0 | (0.0) |  | 1 | (1.8) |  | 1.00 |

* Including Botswana, Madagascar, Sierra Leone, Bolivia, Chile, Cuba, El Salvador, Guyana, Honduras Republic, Nicaragua, Paraguay, Suriname, Cambodia, Maldives, Philippines, Sri Lanka.

^†^ Computed by χ^2^ and Fisher’s exact tests
